# Inhibition of Unc-51-like-kinase is mitoprotective during *Pseudomonas aeruginosa* infection in corneal epithelial cells

**DOI:** 10.1128/msphere.00537-24

**Published:** 2025-01-10

**Authors:** Rajalakshmy Ayilam Ramachandran, Rossella Titone, Joelle T. Abdallah, Mahad Rehman, Mou Cao, Hamid Baniasadi, Danielle M. Robertson

**Affiliations:** 1 Departments of Ophthalmology, The University of Texas Southwestern Medical Center, Dallas, Texas, USA; 2 bioAffinity Technologies, San Antonio, Texas, USA; 3 Department of Biochemistry, The University of Texas Southwestern Medical Center, Dallas, Texas, USA; University of Kentucky College of Medicine, Lexington, Kentucky, USA

**Keywords:** cornea, *Pseudomonas*, infection, autophagy, mitochondria

## Abstract

*Pseudomonas aeruginosa* (PA) is an opportunistic gram-negative pathogen that can infect the cornea, leading to permanent vision loss. Autophagy is a cannibalistic process that drives cytoplasmic components to the lysosome for degradation and/or recycling. Autophagy has been shown to play a key role in the removal of intracellular pathogens and, as such, is an important component of the innate immune response. Autophagy is intimately linked to mitochondria, organelles that mediate energy homeostasis, immune signaling, and cell death. Using a combination of biochemical and imaging approaches, we investigated the effects of PA on autophagy and host cell mitochondria in relation to pro-inflammatory cytokine expression. Using a standard invasive test strain of PA, we show that PA infection triggers dephosphorylation of the mechanistic target of rapamycin in corneal epithelial cells, leading to the induction of autophagy through ULK1/2. This was associated with robust mitochondrial depolarization, changes in mitochondrial ultrastructure, and an increase in IL-6 and IL-8 secretion. PA infection was also associated with an increase in purine metabolism by host cells. Treatment with the ULK1/2 inhibitor, MRT68921, which blocks phagophore formation, attenuated levels of intracellular PA in corneal epithelial cells. Unexpectedly, treatment of cells with MRT68921 blocked PA-induced mitochondrial depolarization and downregulated purine and pyrimidine metabolism. While MRT68921 attenuated the PA-induced increase in IL-6, it further increased IL-8 and neutrophil chemotaxis. This was associated with the nuclear internalization of NF_κ_B. Taken together, these findings highlight a novel mechanism whereby the inhibition of ULK1/2 activity confers mitoprotection during PA infection in corneal epithelial cells.

**IMPORTANCE:**

*Pseudomonas aeruginosa* (PA) is a common pathogen that can cause severe disease in man. In the eye, PA infection can lead to blindness. In this study, we show that PA induces autophagy, a mechanism whereby cells recycle damaged proteins and organelles. PA infection further depolarizes mitochondria, leading to the release of pro-inflammatory mediators. Unexpectedly, the inhibition of ULK1/2, an enzyme involved in the early stages of autophagy, not only inhibits autophagy but enhances mitochondrial polarization. This leads to a reduction in intracellular levels of PA and changes in the inflammatory milieu. Together, these data suggest that the inhibition of ULK1/2 may be mitoprotective in corneal epithelial cells during PA infection.

## INTRODUCTION

*Pseudomonas aeruginosa* (PA) is an opportunistic gram-negative pathogen. In the cornea, acute infection by PA can be visually devastating and lead to permanent vision loss, evisceration, or enucleation of the globe. Prior studies have shown that PA is able to invade corneal epithelial cells (1, 2). This is mediated in part by lipid rafts, sphingolipid and cholesterol-enriched membrane domains (3, 4). Once inside, PA is able to subvert killing and removal by the innate immune system. More recently, studies have shown that PA is able to form membrane blebs in corneal epithelial cells and in the mouse corneal epithelium (5, 6). These blebs are extensions of the plasma membrane that have disassociated from the cytoskeleton. Intracellular replication of PA in corneal epithelial cells has been shown to occur within these membrane blebs (7, 8). Additional work by this same group has shown that bleb formation is mediated by the type III secretion system (T3SS) (7). Loss of function or mutation in key components of the T3SS inhibits bleb formation, leading to the formation of acidic and non-acidic vacuoles that are occupied by PA (7).

Macroautophagy, also known as autophagy, is an intracellular process that involves the catabolic degradation and/or recycling of macromolecules and damaged organelles within lysosomes (9, 10). Autophagy plays fundamental roles in cellular homeostasis and in the pathobiology of disease (11–14). Initiation of autophagy begins with the non-specific engulfment of cytoplasmic constituents by an isolation membrane or phagophore. Continued elongation of this membrane forms the autophagosome. This process is regulated by the activation of the Unc-51-like autophagy activating kinase 1/autophagy-related protein-1 (ULK1/ATG1), a serine/threonine protein kinase that drives the recruitment of autophagy-related proteins and transfer of membrane lipids from the endoplasmic reticulum to the phagophore to promote autophagosomal initiation and maturation (15). Hyperphosphorylation of ULK1 by the mechanistic target of rapamycin (mTOR) pathway inactivates ULK1 activity (16). This in turn leads to an inhibition of autophagy (17). Autophagy has been shown to function as an intracellular innate defense mechanism against microbial pathogens to limit bacterial spread within the host (18, 19). Certain bacteria, however, have evolved to evade or exploit the autophagic process and instead use it as a mechanism to enable intracellular replication and evade the host immune response (20, 21). In the lung, PA infection has been shown to inhibit autophagy (22). Similar to bleb formation in the corneal epithelium, this is mediated by the T3SS.

In addition to mediating macroautophagy, ULK1 has also been shown to translocate to mitochondria (23). There it functions to mediate mitochondria-specific autophagy. Mitochondria are organelles derived from archaebacteria and are well known for their traditional role in energy production. It is now well established that mitochondria also function as major signaling hubs in cells, mediate apoptotic cell death, and drive immune responses (24, 25). In certain cell types, mitochondria are also able to exert antimicrobial effects against pathogens enclosed in phagosomes (26). This occurs through either translocation of mitochondria to the inside of the phagosome or the release of mitochondrial-derived vesicles (26, 27). In the present study, we investigated the ability of PA to induce autophagy in corneal epithelial cells. We further examined mitochondrial changes that occur during infection. Importantly, we show that PA not only exploits autophagy in corneal epithelial cells, but we provide evidence supporting a novel role for ULK1/2 in mediating the mitochondrial response during infection in the cornea.

## RESULTS

### PA infection induces autophagy in corneal epithelial cells through mTOR

**Fig 1:**
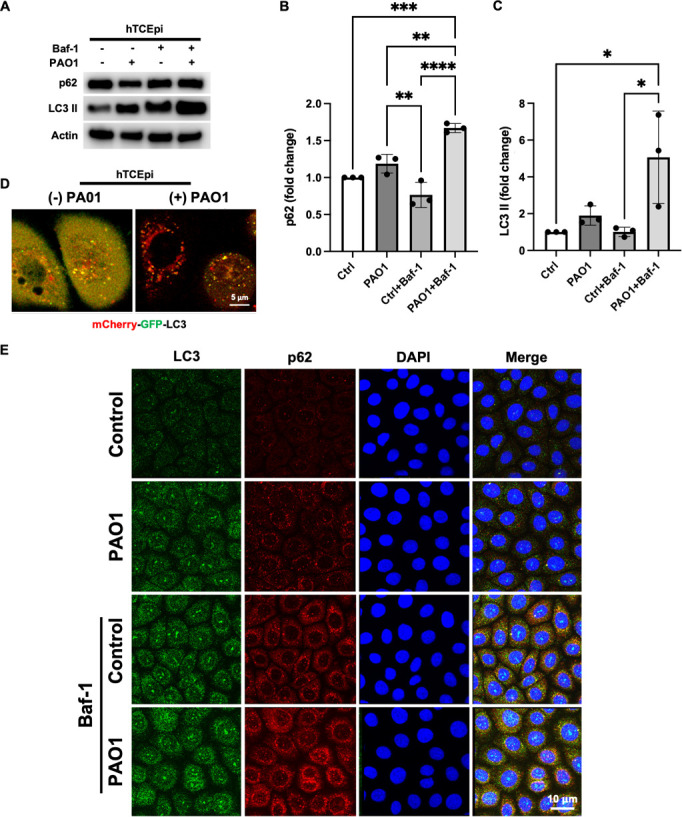
PA induces autophagy in corneal epithelial cells. hTCEpi cells were treated with bafilomycin-1 (Baf-1) for 1 hour. Cells were then infected with 10^6^ CFU/mL of PAO1 for 2 hours with or without Baf-1. (**A**) Immunoblotting for p62 and LC3-II. Actin was used as loading control. (**B and C**) Quantification of p62 and LC3-II with or without Baf-1. Data normalized to the untreated control. One-way analysis of variance (ANOVA) with Tukey’s post hoc multiple comparison test, *P < 0.05, **P < 0.01, ***P < 0.001, and ****P < 0.0001. (**D**) Cells were transfected with an mCherry-GFP-LC3 expression plasmid. Yellow puncta indicate the presence of LC3-II positive organelles (autophagosomes), and red puncta indicate the presence of LC3-II positive organelles that have fused with lysosomes (autophagolysosomes). Scale bar: 5 µm. (**E**) Immunofluorescence for p62 and LC3. Scale bar: 10 µm. Images representative of three repeated experiments.

To determine whether PA infection induces autophagy in human-telomerase-immortalized corneal epithelial (hTCEpi) cells, we first measured the expression of the autophagy markers p62 and lipidated LC3 by immunoblotting. To quantify autophagic flux, the autophagy inhibitor bafilomycin (Baf-1) was used to block the fusion of autophagosomes with acidic lysosomes (Fig. 1A; Fig. S1). In the presence of Baf-1, both p62 and LC3 II accumulated in PA-infected cells compared to non-infected cells (Fig. 1A through C). Similar results were found in primary cultured human corneal epithelial cells (HCECs, Fig. S2). To confirm an increase in autophagy, hTCEpi cells were transfected with a pH-sensitive expression plasmid, mCherry-GFP-LC3. Using a live cell imaging approach, we observed an increase in LC3 positive organelles that had undergone fusion with lysosomes (red puncta) in hTCEpi cells (Fig. 1D). Consistent with this, immunofluorescent staining for LC3 and p62 also showed an increase in expression after PA infection that was further increased after treatment with Baf-1 (Fig. 1E).

**Fig 2:**
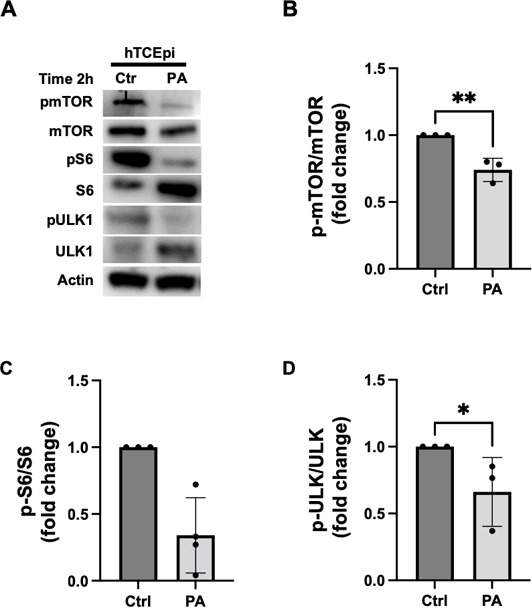
PA downregulates mTOR/S6/ULK1 signaling in corneal epithelial cells. hTCEpi cells were treated with bafilomycin-1 (Baf-1) for 1 hour. Cells were then infected with 10^6^ CFU/mL of PAO1 for 2 hours with or without Baf-1. (**A**) Immunoblots for hTCEpi cells showed a decrease in phosphorylation of mTOR, S6, and ULK. Total protein levels of mTOR, S6, and ULK were used as loading controls. (**B–D**) Quantification of phosphorylated protein. Data normalized to the control. Two-tailed *t*-test, *P < 0.05 and **P < 0.01.

Since activation of mTOR is known to inhibit autophagy, we next investigated the effects of PA on the regulation of mTOR signaling by measuring the phosphorylation levels of mTOR and its downstream substrates S6 and ULK1 by immunoblotting (Fig. 2A through D). In hTCEpi cells, phosphorylated levels of mTOR, S6, and ULK1 were all decreased 2 hours post infection (Fig. 2A). Quantification of the immunoblots confirmed the decrease in phosphorylated levels of mTOR, S6, and ULK1 proteins (Fig. 2B through D). These data indicate that PA downregulates the mTOR pathway in corneal epithelial cells to promote autophagy. We next tested the effects of PA infection on mTOR signaling using HCECs. Consistent with our cell line findings, components of the mTOR pathway were dephosphorylated 2 hours post infection in primary cultures (Fig. S2). As an additional control, the experiment was repeated with or without viable or heat-killed PA (Ϯ). Indeed, heat-killed PA failed to induce the dephosphorylation of mTOR in corneal epithelial cells (Fig. S3).

### Perturbation of autophagy alters intracellular levels of PA

**Fig 3:**
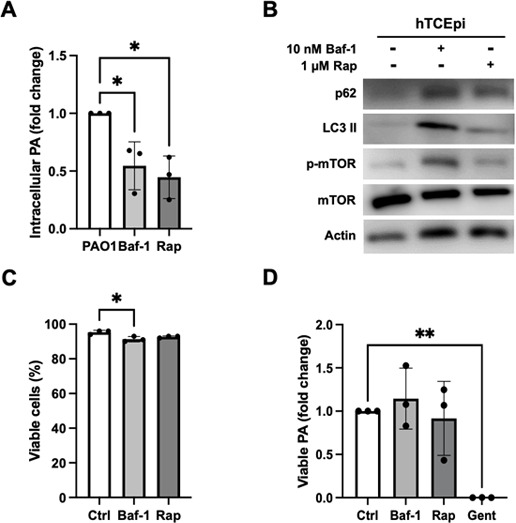
Disruption of PA-induced autophagy reduces intracellular levels of PA. hTCEpi cells were treated with bafilomycin-1 (Baf-1) or rapamycin (Rap) for 1 hour. Cells were then infected with 10^6^ CFU/mL of PAO1 for 2 hours with or without Baf-1 or Rap. (**A**) Gentamicin protection assay. Treatment with Baf-1 and Rap both decreased intracellular levels of PA. (**B**) Immunoblots confirming the inhibition and stimulation of autophagy by treatment with Baf-1 and Rap, respectively. Actin was used as a loading control. (**C and D**) Neither Baf-1 nor Rap induced any measurable level of cytotoxicity in hTCEpi cells (**C**) or reduced PA viability (**D**). Gentamicin was used as a positive control. Data normalized to the untreated control. One-way ANOVA with Tukey’s multiple comparisons test, *P < 0.05 and **P < 0.01.

To determine the effect of autophagy on levels of intracellular PA, we next performed a gentamycin protection assay. Prior to infection, cells were treated with bafilomycin (Baf-1) or rapamycin (Rap) to inhibit or stimulate autophagy, respectively. Treatment with either Baf-1 or Rap showed a decrease in the amount of intracellular PA (Fig. 3A). The induction or inhibition of autophagy was confirmed by immunoblotting for p62 and LC3 II (Fig. 3B). Despite the decrease in internalized PA with both inhibitors, neither bafilomycin nor rapamycin adversely impacted hTCEpi cell viability (Fig. 3C) or PA viability (Fig. 3D). These data suggest that any perturbation of autophagy during infection reduces intracellular levels of PA in corneal epithelial cells.

### ULK1/2 inhibition decreases intracellular PA in hTCEpi cells

**Fig 4:**
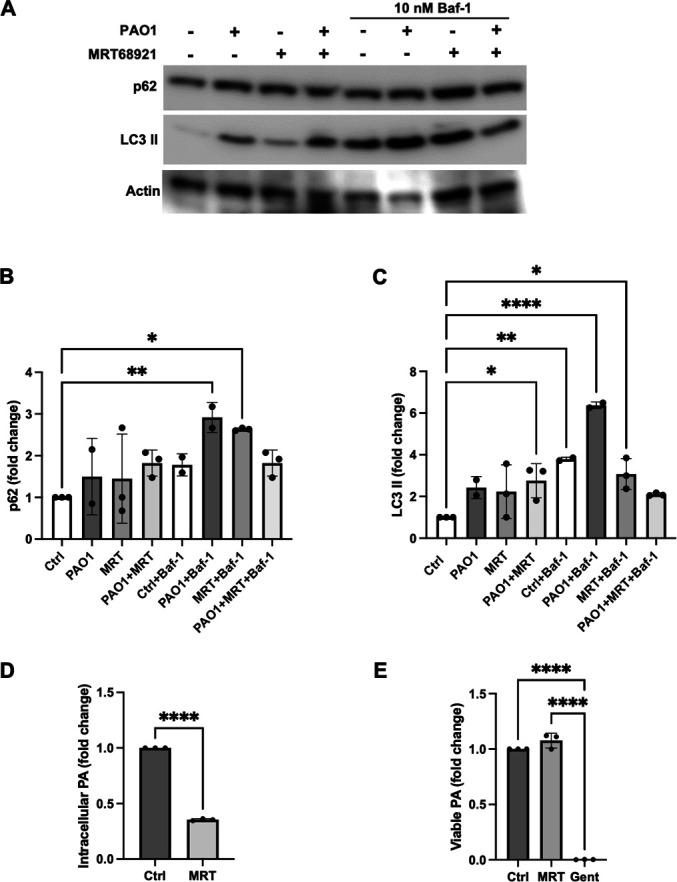
The inhibition of ULK1/2 attenuates levels of intracellular PA in corneal epithelial cells. hTCEpi cells were treated with MRT68921 for 1 hour prior to inoculation with 10^6^ CFU/mL of PAO1 for 2 hours. (**A**) Immunoblotting for p62 and LC3-II. Actin was used as loading control. (**B and C**) Quantification of immunoblots. Treatment with the ULK1/2 inhibitor MRT68921 inhibited PA-induced p62 and LC3-II accumulation. One-way ANOVA with Tukey’s post hoc multiple comparison test. (**F**) Gentamicin protection assay. Treatment with MRT68921 reduced intracellular levels of PA. Two-tailed *t*-test. (**G**) MRT68921 had no effect on PA viability. Gentamicin was used as a positive control. One-way ANOVA with Tukey’s post hoc multiple comparison test, *P < 0.05, **P < 0.01, and ****P < 0.0001. Data normalized to the untreated control.

ULK1/2 is an integral protein that functions in the initiation of autophagy by mediating the formation of the phagophore and promoting autophagosome maturation (28, 29). To further examine the effects of autophagy on PA invasion, we used the ULK1/2 inhibitor MRT68921 (Fig. S1) (29). In hTCEpi cells, we again saw that PA infection in the presence of Baf-1 increased levels of p62 and LC3 II (Fig. 4A through C). The addition of MRT68921 in cells treated with Baf-1 prior to infection, however, reduced p62 and LC3 II back to control levels. To determine whether the inhibition of ULK1/2 altered intracellular PA survival, we used a gentamicin protection assay. Consistent with the effects of rapamycin and bafilomycin, MRT68921 similarly reduced intracellular levels of PA to approximately one-third of control levels (Fig. 4D). As an additional control, we confirmed that the inhibitor had no effect on PA viability (Fig. 4E). These findings again demonstrate that the inhibition of autophagy decreases intracellular levels of PA in corneal epithelial cells.

**Fig 5:**
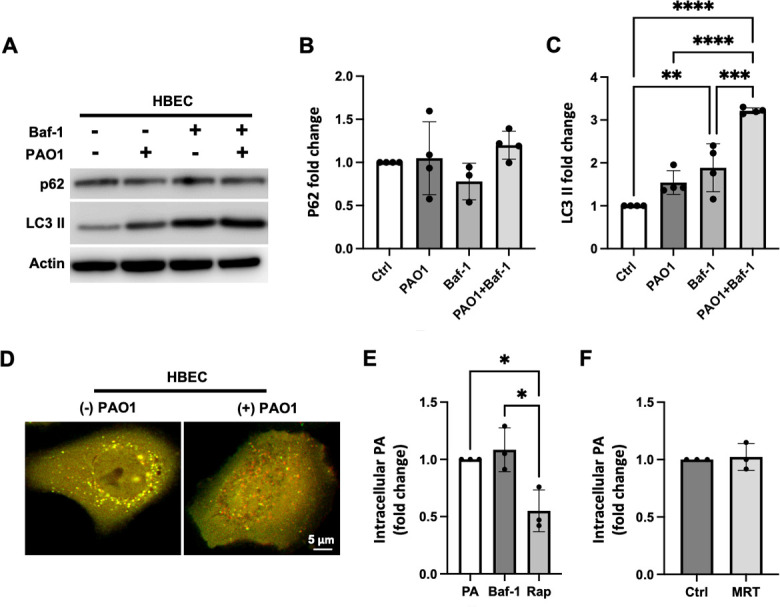
There was minimal induction of autophagy in bronchial epithelial cells. (**A–E**) HBECs were treated with bafilomycin-1 (Baf-1) or rapamycin (Rap) for 1 hour. Cells were then infected with 10^6^ CFU/mL of PAO1 for 2 hours with or without Baf-1 or rap. (**A**) Immunoblotting for p62 and LC3-II in HBECs. Actin was used as loading control. (B and C) Quantification of immunoblots. p62 was unchanged after infection by PA. There was a small but significant increase in LC3-II in cells treated with Baf-1. One-way ANOVA with Tukey’s post hoc multiple comparison test. (**D**) mCherry-GFP-LC3 expression. Yellow puncta indicate the presence of LC3-II positive organelles (autophagosomes), and red puncta indicate the presence of LC3-II positive organelles that have fused with lysosomes (autophagolysosomes). Scale bar: 5 µm. Images representative of three repeated experiments. (**E and F**) Gentamicin protection assay. (**E**) hTCEpi cells were treated with MRT68921 for 1 hour prior to inoculation with 10^6^ CFU/mL of PAO1 for 2 hours. Unlike hTCEpi cells, treatment with Baf-1 did not alter intracellular levels of PA. One-way ANOVA with Tukey’s post hoc multiple comparison test. (**F**) Intracellular levels of PA were unaffected by treatment with the ULK1/2 inhibitor, MRT68921. Two-tailed *t*-test. *P < 0.05, **P < 0.01, ***P < 0.001, and ****P < 0.0001. Data normalized to the untreated control.

In the lung, PA has been previously shown to inhibit autophagy 6 hours post infection (22). As a control, we next investigated the ability of PA to induce autophagy in human-telomerized bronchial epithelial cells (HBECs). Two hours post infection, we were unable to detect a change in p62 expression in the presence of Baf-1 (Fig. 5A and B). There was, however, a small but significant increase in the expression of LC3 II (Fig. 5A and C). Using the mCherry-GFP-LC3 reporter plasmid, some red puncta were evident in PA-infected cells (Fig. 5D), suggesting a mild induction of autophagy. Similar to corneal epithelial cells, the induction of autophagy in HBECs using rapamycin decreased intracellular levels of PA (Fig. 5E). However, intracellular levels of PA in HBECs were unaffected by treatment with Baf-1. Consistent with this, treatment with the ULK1/2 inhibitor, MRT68921, also had no effect on intracellular levels of PA (Fig. 5F). These data demonstrate that in bronchial epithelial cells, there is little induction of autophagic flux during the first 2 hours of infection, and blocking autophagy has no effect on intracellular levels of PA.

### ULK1/2 inhibition is mitoprotective during PA infection in corneal epithelial cells

**Fig 6:**
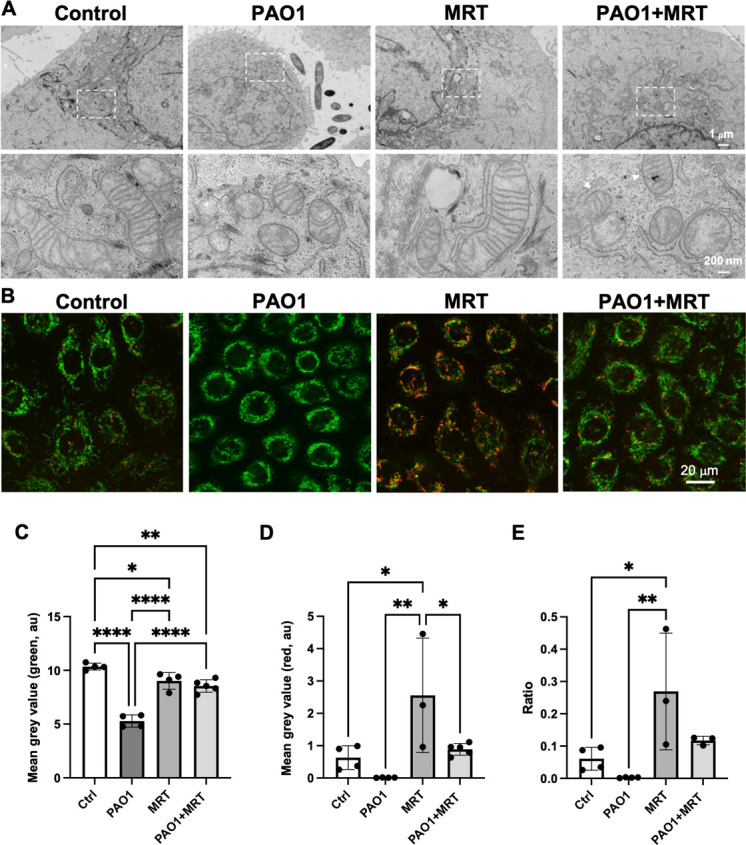
PA infection alters mitochondrial structure and physiology in corneal epithelial cells. hTCEpi cells were treated with MRT68921 for 1 hour prior to inoculation with 10^6^ CFU/mL of PAO1 for 2 hours. (**A**) Transmission electron microscopy. Compared to control cells, PA-infected cells had smaller mitochondria with altered cristae morphology. PA-infected cells treated with MRT68921 had small mitochondria, but there was evidence of intact lamellar cristae (arrows). Lower panels are zoomed images of the area shown in dotted rectangle. Scale bar: 1 µm in upper panels and 200 nm in lower panels. (**B**) JC-1 staining to assess mitochondrial polarization. Mitochondrial areas of low polarization shown in green and areas of high polarization shown in red. PA-infected cells are depolarized and have abnormal mitochondrial morphology. PA-infected cells treated with MRT68921 are polarized similar to control cells with partial restoration of the tubular mitochondrial network. Scale bar: 20 µm. (**C and D**) Quantification of green fluorescence (**C**), red fluorescence (**D**), and the ratio of red to green (**E**). One-way ANOVA, Tukey’s post hoc multiple comparison test. *P < 0.05, **P < 0.01, ***P < 0.001, and ****P < 0.0001. Images representative of three repeated experiments.

In addition to macroautophagy, ULK1 has been shown to translocate to damaged mitochondria to promote their removal (23). Several types of invasive bacteria have also been shown to target mitochondria; however, the ability of PA to target and fragment mitochondria has not been previously shown (30). To investigate whether ULK1/2 inhibition affected mitochondria, hTCEpi cells were imaged using transmission electron microscopy. As shown in Fig. 6, mitochondria in PA-infected cells were smaller and rounder, with less organized cristae compared to non-infected cells (Fig. 6A). In cells treated with the ULK1/2 inhibitor, mitochondria were similar to control cells with well-defined lamellar cristae morphology. In PA-infected cells treated with MRT68921, mitochondria were still small and round, but a subset of mitochondria demonstrated enhanced lamellar architecture. To further examine the effects of the ULK1/2 inhibitor on mitochondria, cells were stained with the membrane-permeable cationic dye, JC-1. JC-1 accumulates in polarized mitochondria. In areas of low polarization, JC-1 is present as a monomer and fluorescent green. In areas of high membrane polarization, JC-1 forms aggregates that emit red fluorescence. In our model, JC-1 staining revealed a sigificant loss of mitochondrial membrane polarization in cells infected with PA (Fig. 6B). In addition, mitochondria no longer exhibited a tubular network as seen in control cells but were shortened and more perinuclear. Unexpectedly, treatment of non-infected cells with MRT68921 increased polarization compared to the non-infected control. This mitoprotective effect was evident during infection, where the inhibitor blocked the PA-induced loss of mitochondrial membrane depolarization. In these cells, JC-1 staining was similar to control cells. Quantification of red and green fluorescence is shown in Fig. 6C and D. The red:green ratio was significantly decreased in PA-infected cells (Fig. 6E). Treatment with MRT68921 prevented this decrease. Collectively, these data suggest that the inhibition of ULK1/2 may be mitoprotective to corneal epithelial cells during infection.

### ULK1/2 inhibition restores host cell metabolism

**Fig 7:**
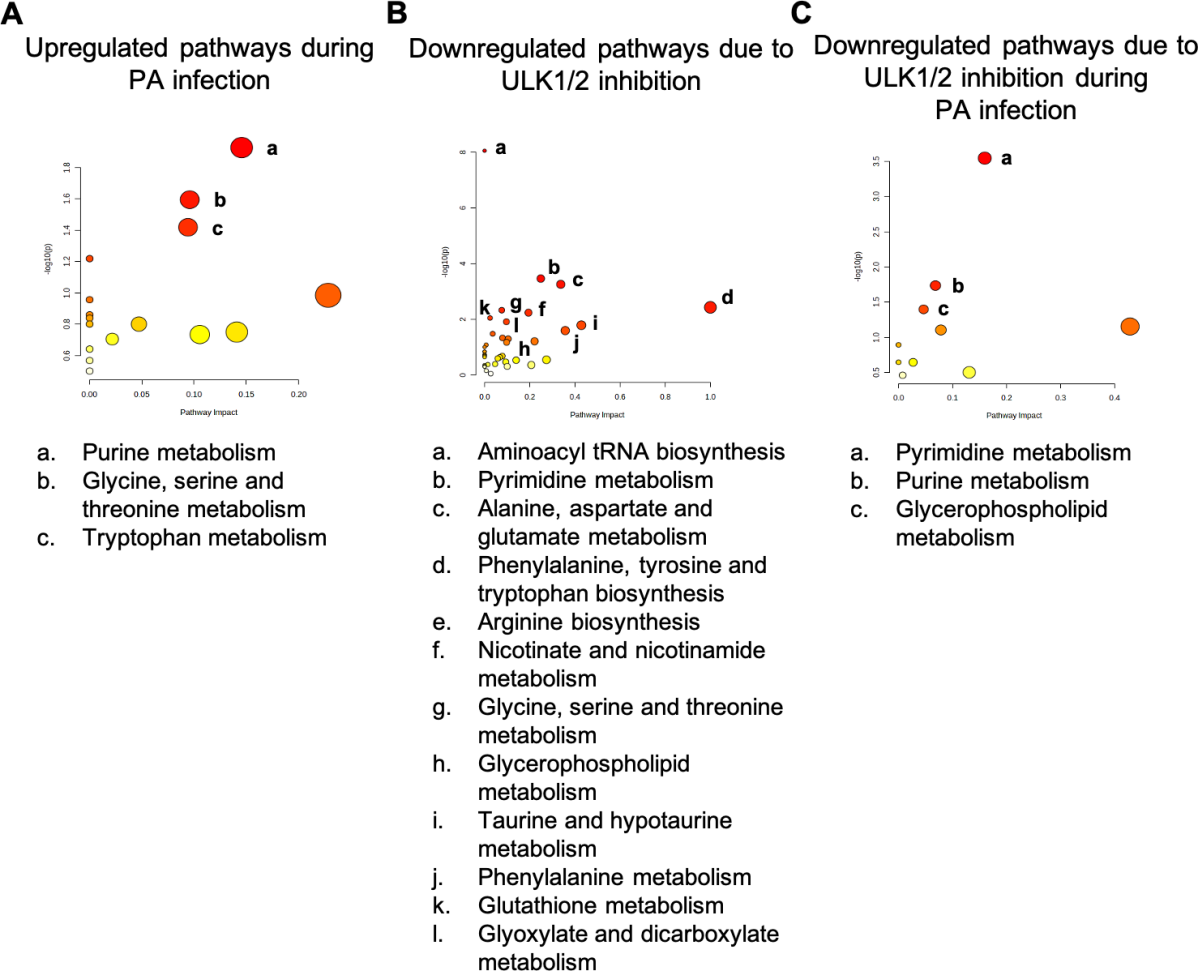
The PA-mediated increase in purine metabolism is attenuated by treatment with MRT6821. hTCEpi cells were treated with MRT68921 for 1 hour prior to inoculation with 10^6^ CFU/mL of PAO1 for 2 hours. (**A**) Pathway impact analysis showed an increase in purine, glycine, serine, threonine, and tryptophan metabolism in PA-infected cells. (**B**) Glycine, serine, and threonine metabolism were decreased in non-infected cells treated with MRT68921. (**C**) Treatment with MRT68921 decreased pyrimidine, purine, and glycerophospholipid metabolism in PA-infected cells. The size of the circles indicates impact, with larger circles representing the most impacted pathways. The color of the circles indicates significance with red representing a lower *P* value and yellow representing a higher *P* value.

Due to the dramatic effect of ULK1/2 on host cell mitochondria, we next sought to examine the effects of ULK1/2 inhibition on host cell metabolism using an untargeted metabolomics approach. Metabolomics was performed 2 hours post infection. Infection of hTCEpi cells with PA led to an upregulation of purine, glycine, serine, threonine, and tryptophan metabolism (Fig. 7A). In bacteria, mutants deficient in purine metabolism have been shown to have a limited replicative capacity (31). To overcome this, certain anaerobic bacteria in the gut are able to utilize host purine metabolism as a source of carbon and nitrogen (32). Thus, the increase in purine metabolism in host cells may have important implications in terms of nutrition for intracellular PA. The effects of treatment with MRT68921 are shown in Fig. 7B. During PA infection, the inhibition of ULK1/2 downregulated both purine and pyrimidine metabolism (Fig. 7C). This has the potential to limit nutrient availability and the requisite supply of nucleotides needed by PA for replication. Whether these decreases were a consequence of fewer PA inside the cell or due to direct effects on PA is unknown but represents a future avenue of study. Since PA was intracellular in infected cells, as an additional control, we also performed metabolomics on a PA-only group to differentiate between host and PA metabolites. Importantly, metabolites in the PA-only group failed to reach the detection threshold of the mass spectrometer, confirming that the metabolites shown in Fig. 7 were exclusive to host cells.

### ULK1/2 inhibition alters pro-inflammatory cytokine production

**Fig 8:**
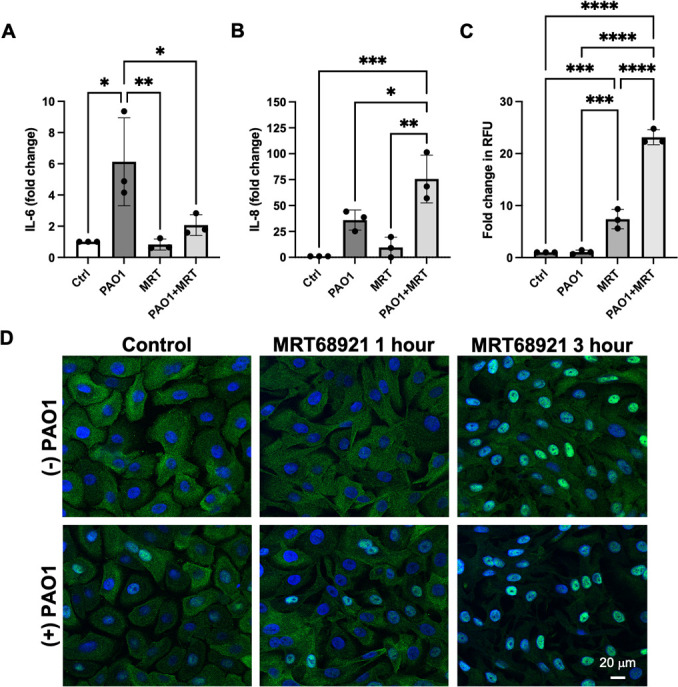
ULK1/2 inhibition alters inflammatory cytokine secretion and neutrophil chemotaxis during PA infection in corneal epithelial cells. (**A–C**) hTCEpi cells were inoculated with 10^6^ CFU/mL of PAO1 for 6 hours with or without MRT68921. (**A**) MRT68921 inhibited IL-6 secretion by PA-infected cells. (**B**) MRT68921 increased IL-8 secretion in PA-infected cells. (**C**) The increase in IL-8 secretion paralleled an increase in neutrophil chemotaxis. Data normalized to the untreated control, *n* = 3. One-way ANOVA, Tukey’s post hoc multiple comparisons test, *P < 0.05, **P < 0.01, ***P < 0.001, and ****P < 0.0001. (**D**) Immunostaining for NF_κ_B p65. MRT68921 increased nuclear translocation. MRT68921 1 hour indicates 1 hour of incubation with MRT68921 followed by a 2-hour incubation with PA. MRT68921 3 hour indicates 1 hour of incubation with MRT68921 followed by a 2-hour incubation with PA and MRT68921. Scale bar: 20 µm.

It is well established that mitochondrial stress triggers the release of mitochondrial DNA (mtDNA), a potent pro-inflammatory stimulus (33). Here, we tested the ability of PA to induce secretion of the pro-inflammatory cytokines, IL-6, and IL-8 (Fig. 8A and B). While PA increased IL-6 secretion by sixfold, IL-8 secretion was increased over 35-fold. Consistent with the improvement in mitochondrial ultrastructure, treatment with the ULK1/2 inhibitor attenuated IL-6 levels. IL-8 levels, however, were further increased. Since IL-8 is a major chemotactic factor for neutrophils, we used a Boyden chamber assay to examine neutrophil migration (Fig. 8C). Cell culture supernatants from non-infected MRT68921-treated cells showed a modest increase in neutrophil chemotaxis compared to control. In PA-infected cells treated with MRT68921, neutrophil migration was increased threefold over the non-infected MRT68921 treatment condition. To further investigate the effects of MRT68921 on hTCEpi cells, we next evaluated the nuclear translocation of the NF-κB subunit p65, which is known to promote IL-8 expression. As shown in Fig. 8D, treatment with the ULK1/2 inhibitor, MRT68921, potentiated nuclear translocation in the presence and absence of PA.

## DISCUSSION

The first key finding in this study is the demonstration that infection by PA induces autophagy in corneal epithelial cells. This finding is supported by our data in Fig. 2 that shows a reduction in mTOR signaling and the accumulation of p62 and lipidated LC3 in the presence of the V-ATPase bafilomycin. Consistent with this, we also found changes in the mTOR signaling pathway. Mohankumar and colleagues previously investigated the potential for PA to induce autophagy in a serum-based corneal epithelial cell model (34). Using nine different clinical isolates of PA and PAO1 T3SS mutants, they too demonstrated that PA was able to promote autophagy, and this observation was dependent on the T3SS (34). A limitation of that prior work, however, was the use of a human corneal epithelial cell line that does not express cornea-specific differentiation markers (35). Given the importance of cell type specificity in autophagy, we have now addressed that limitation in the current study.

In mammalian cells, it is well established that there exists an optimal level of autophagy to support the health of the cell (36). Too little autophagy or excessive autophagy is associated with cell death. As illustrated in Fig. 3, our data point toward a similar paradigm in which there is an optimal level of autophagy during PA infection. This finding was exclusive to corneal epithelial cells. These data suggest that PA may be exploiting autophagy during the early stages of infection in corneal epithelial cells to evade host responses but may be overwhelmed by the increased autophagic response from rapamycin. An alternative hypothesis is that rapamycin is somehow able to fend off or alter virulence factors produced by PA. Indeed, rapamycin has been shown to impede the toxic effects of the cytolethal-distending toxin produced by *Campylobacter jejuni* (37). This may explain the similar findings that were observed in bronchial epithelial cells in our study and in work by Junkins and colleagues who also found that the rapamycin-induced increase in autophagy was associated with a reduction in intracellular PA (38). Comparable findings were also observed in alveolar macrophages (39).

While rapamycin increased PA clearance in both corneal and bronchial epithelial cells, the inhibition of autophagy had differing effects. In our hands, there was very minimal activation of autophagy in bronchial epithelial cells 2 hours post infection. Consistent with this, treatment with bafilomycin failed to reduce intracellular levels of PA in this cell type (Fig. 5) (37). Rao and colleagues previously showed that PA uses the T3SS to inhibit autophagy in bronchial epithelial cells (22). In that study, autophagy was investigated 6 hours post infection. This finding, together with the absence of a significant autophagic response in bronchial epithelial cells at the 2-hour time point, may explain why we failed to see an effect with bafilomycin. Regardless, additional study is needed to determine whether the inhibition or activation of autophagy exerts these effects via the same or different mechanisms.

Due to the ability of bafilomycin to reduce intracellular levels of PA, we next hypothesized that the inhibition of ULK1/2, an integral kinase involved in the initiation of autophagy, would similarly reduce intracellular levels of PA in corneal epithelial cells by preventing the induction of autophagy. Since ULK1/2 activity is phosphorylation dependent, whereby dephosphorylation leads to activation of ULK1/2 and generation of the isolation membrane and autophagosome maturation, we used MRT68921, a known inhibitor of ULK activity (28, 29, 40). Indeed, the inhibition of ULK1 reduced intracellular levels of PA. Like bafilomycin, this finding was specific to corneal cells. ULK1/2 inhibition also blunted the PA-induced increase in host cell purine metabolism and reduced pyrimidine metabolism. Host cell purine and pyrimidine metabolism likely provide key molecules that are used by PA to support replication within their intracellular niche.

The most robust effect of ULK1/2 inhibition, however, was on host cell mitochondria (Fig. 6). We initially explored the effects of ULK1/2 inhibition on mitochondria due to the reported ability of ULK1 to translocate to damaged mitochondria in mouse embryonic fibroblasts (23). Unexpectedly, we found that treatment of non-infected corneal epithelial cells resulted in mitochondrial hyperpolarization compared to control cells. More importantly, and the second key finding of this study, is that ULK1/2 inhibition was able to preserve mitochondrial polarization during infection. Together, these data suggest that the inhibition of ULK1/2-mediated autophagy is mitoprotective in the corneal epithelium through an unknown mechanism. The observed reciprocal relationship between ULK activity and mitochondrial polarization during infection (activity increased) and following treatment with the inhibitor (no activity) lessens the likelihood that the mitoprotective role of MRT68921 is due to off-target effects. Interestingly, despite the effect on polarization, electron microscopy did not show retention of mitochondrial ultrastructure following ULK1/2 inhibition. Mitochondria remained small and rounded. Instead, there was a partial enhancement of the lamellar cristae architecture in PA-infected cells. Since cristae are the site of oxidative phosphorylation, this enhancement in the lamellar architecture may translate into an increased capacity to generate ATP and protect against stress.

In addition to energy generation, mitochondria are now widely recognized as key mediators of innate immune responses (33). This includes the release of pro-inflammatory mtDNA leading to the activation of the cGAS/STING pathway (33, 41). Imbalances in pyrimidine metabolism, as seen in this study, have also been linked to changes in mitochondria and mtDNA release (42). The release of mtDNA is a known stimulus for IL-6 and IL-8 secretion. Here, we found that PA infection upregulated the secretion of both IL-6 and IL-8 (Fig. 8). Stabilization of the inner mitochondrial membrane by ULK1/2 inhibition, however, was only associated with a reduction in IL-6. In contrast, IL-8 was increased, further amplifying the chemotactic stimulus for neutrophils. The use of small interfering RNA (siRNA) and a chemical ULK1/2 inhibitor, SBI0206965, has been shown to inhibit IL-6 and IL-8 release by keratinocytes in psoriatic skin (43). The mechanism driving the selective impairment in IL-6 compared to IL-8 in our model is unknown. Our data suggest that MRT68921 may be driving the nuclear localization of NF_κ_B in corneal epithelial cells, and this may potentiate the increase in IL-8 secretion in these cells. Despite this increase, however, levels of IL-8 in cell culture supernatants from non-infected cells treated with the ULK inhibitor remained low, suggesting that some other cytokine and/or pro-inflammatory mediator is driving neutrophil migration.

In summary, these data indicate that PA induces autophagy in corneal epithelial cells, and autophagy is important for invasion. The reduction in intracellular PA with both the induction and the inhibition of autophagy likely occurs through disparate mechanisms. Further study is needed to determine whether these changes are due to bacterial invasion or intracellular survival. Similarly, while the inhibition of ULK1/2 impaired autophagy, it appeared to be mitoprotective during PA infection. Mitochondria have been shown to play important roles in host defense in other disease models (44). Thus, both of these pathways require more work, including longer time points to evaluate PA replication and survival, in order to tease out the potential contributory role of each during infection.

## MATERIALS AND METHODS

### Cell lines and primary cultures

The hTCEpi cell line used in this study was previously developed and characterized by our laboratory (45). The HBECs were a generous gift from Dr. Jerry Shay in the Department of Cell Biology at UT Southwestern Medical Center (46). hTCEpi cells and HBECs were cultured in serum-free keratinocyte basal media containing 0.15 mM calcium and 6.0 mM glucose with supplements at 37°C and 5% CO_2_ (KGM, PromoCell, Germany). For routine maintenance, cell culture media contained 10% Pen/Strep/amphotericin B. Primary cultures of HCECs were derived from organ donors. Donor corneas were obtained from our on campus eye bank (Transplant Services Center, UT Southwestern Medical Center, Dallas, TX), and cultures were generated as previously reported (45). Briefly, corneas were digested in 5 U/mL of dispase (Invitrogen, Carlsbad, CA) overnight at 4°C. Intact epithelial cell sheets were carefully removed and subjected to a second digestion in 5 U/mL dispase for 2 hours at 37°C. Cells were then separated by gentle pipetting and seeded onto plastic tissue culture dishes coated with Type IV collagen (Biocoat, BD Biosciences, San Jose, CA). HCECs were cultured in CnT20 cell culture media enriched for progenitor cell culture for 10–15 days (Zen Bio, Research Triangle Park, NC). After their first passage, HCECs were then transitioned to serum-free keratinocyte basal media containing 0.15 mM calcium with supplements at 37°C and 5% CO_2_ (KGM2, PromoCell, Germany). For all experiments, cells were cultured in keratinocyte growth media without antibiotics. For the neutrophil experiments, HL-60 cells were purchased from ATCC (Manassas, VA) and routinely cultured in Isocove’s modified Dulbecco’s medium (IMDM; ATCC) with 20% fetal bovine serum (Sigma-Aldrich, St. Louis, MO) and 10% Pen/Strep/amphotericin B. To differentiate cells into neutrophil-like cells, 1.3% dimethyl sulfoxide (DMSO) was added to the cell culture media for 5 days. Cells were maintained at 37°C and 5% CO_2_.

### Bacteria

For all experiments, hTCEpi cells and HBECs were inoculated with a concentration of 10^6^ CFU/mL of a standard invasive test strain of PA, PAO1 (gift of Dr. Suzanne Fleiszig, UC Berkeley) for 2 or 6 hours (47). PAO1 was cultured overnight to log growth phase. PA was resuspended in phosphate-buffered saline (PBS), and the optical density was adjusted to 1 × 10^8^ CFU/mL. Concentration was measured using a SmartSpec Plus spectrophotometer (Bio-rad, Hercules, CA). The bacterial suspension was diluted to 1 × 10^6^ CFU/mL for all experiments. Inoculums were confirmed by serial dilution in PBS and plating on tryptic soy agar. All samples were plated in triplicate for analysis of colony counts. PAO1 is a risk group 2 pathogen; therefore, all biosafety level 2 precautions were used during the experiments. As an additional control to confirm the effects were due to viable bacteria, heat-killed bacteria were used after incubation at 60°C for 30 minutes. Loss of viability was confirmed by colony counts. The potential bactericidal effects of all inhibitors were tested. For these assays, PAO1 at log growth phase was used. The bacterial suspension was diluted to 1 × 10^6^ CFU/mL and treated with either 10 nM bafilomycin A1 or 1 µM rapamycin for 3 hours in static culture at 37°C. For all other experiments, cells were treated with bafilomycin A1 or rapamycin for 1 hour, followed by PA inoculation with or without each inhibitor for 2 hours. Cultures were then serially diluted in PBS, plated on tryptic soy agar in triplicate, and incubated overnight for colony count determination. The antibiotic gentamicin (Sigma, St. Louis, MO) was used as antibacterial positive control at a concentration of 200 µg/mL.

### SDS PAGE and immunoblotting

hTCEpi cells and HBECs were plated in six-well culture plates and allowed to adhere overnight. Cells were inoculated with or without PAO1, and after that, cells were lysed using lysis buffer containing 50 mM Tris-HCl (pH 7.5), 150 mM NaCl, 1% Triton X-100, 1 mM EDTA, and a protease and phosphatase inhibitor cocktail (Thermo Fisher, Rockford, IL) on ice for 10 minutes. Some samples were pre-treated with either bafilomycin A1 (Baf-1, Sigma, St. Louis, MO) or the ULK 1/2 inhibitor MRT68921 (MedChemExpress, Monmouth Junction, NJ). Lysates were then centrifuged for 10 minutes at 10,000 × *g* at 4°C in a microcentrifuge (BioRad, Hercules, CA). Supernatants were removed and boiled for 5 minutes in 2× sample buffer (pH 6.8), containing 65.8 mM Tris-HCl, 26.3% (wt/vol) glycerol, 2.1% SDS, 5.0% β-mercaptoethanol, and 0.01% bromophenol blue (Bio-rad, Hercules, CA). Samples were resolved on a 4%–15% precast linear gradient polyacrylamide gel (Bio-rad, Hercules, CA) and transferred to a polyvinyl difluoride membrane (Bio-rad, Hercules, CA). Membranes were blocked in 5% non-fat milk in PBS containing 0.1% Tween 20 (Bio-rad, Hercules, CA) for 1 hour at room temperature and incubated in primary antibody overnight at 4°C. The following primary antibodies were used: mTOR (#2983), p-mTOR (#2971), ULK1 (#8054), pULK1 (#6888), s6 ribosomal protein (#2217), and p-S6 ribosomal protein (#4856, Cell Signaling, Danvers, MA); β-actin (#sc-47778, Santa Cruz, CA); LC3B (#L7543, Sigma, St. Louis, MO); and p62 (#H00008878, Novusbio, Littleton, CO). Membranes were washed and incubated for 1 hour with an anti-mouse or anti-rabbit secondary antibody (Santa Cruz, CA). Proteins were visualized using enhanced chemiluminescence (ECL) Prime Detection Reagent (Thermo Fisher, Rockford, IL) and imaged on an Amersham Imager 600 (Amersham Biosciences, Piscataway, NJ). β-Actin was used as a loading control. Immunoblots were quantified using ImageQuant TL Toolbox v8.1 software (Amersham Bioscience, Piscataway, NJ). Images were analyzed using area and profile-based tools. The bands of all proteins were normalized using actin. In the case of phosphorylated proteins, the respective total protein was used for normalization.

### Immunofluorescence

For immunofluorescent labeling, hTCEpi cells were seeded onto 35 mm glass coverslip bottom dishes (MatTek Corporation, Ashland, MA) at 70% confluence and allowed to adhere overnight. The medium was then removed and replaced with fresh KGM, and cells were grown to confluence. For LC3 and p62 immunofluorescence, cells were inoculated with 10^6^ CFU/mL for 2 hours with or without 10 nM Baf-1. For NFκB immunofluorescence, cells were treated with MRT68921 for 1 hour. Then medium was changed and replaced with 10^6^ CFU/mL with or without MRT68921 for an additional 2 hours. Control cells were infected with PA for 2 hours. After all treatments, cells were then washed with cold PBS and fixed for 10 minutes using 1% paraformaldehyde in PBS (Electron Microscopy Sciences, Fort Washington, PA) at room temperature. Cells were again washed with PBS and permeabilized for 10 minutes with 0.1% Triton X-100 in PBS. After an additional three washes in PBS, cells were blocked for 30 minutes using 0.5% bovine serum albumin in PBS (Sigma, St. Louis, MO) at room temperature. Cells were incubated overnight at 4°C in primary antibody diluted in 0.1% bovine serum albumin (Sigma, St. Louis, MO). The following primary antibodies were used: p62 (#H00008878, Novusbio, Littleton, CO), LC3B (#L7543, Sigma, St. Louis, MO), and NFκB p65 (#8242S, Cell Signaling, Danvers, MA). The next morning, each dish was washed with PBS and incubated with an appropriate secondary antibody: anti-rabbit IgG secondary antibody conjugated to Alexa Fluor 488 or Alexa Fluor 555 (Cell Signaling, Danvers, MA) at room temperature. Prolong gold anti-fade reagent with DAPI was used to label the nucleus (Invitrogen, Carlsbad, CA). A Leica SP8 laser scanning confocal microscope with a 63× oil objective was used to acquire images (Leica Microsystems, Heidelberg, Germany). Images were sequentially scanned in order to avoid spectral crosstalk between channels.

### Mitochondrial polarization

To quantify mitochondrial polarization, hTCEpi cells were seeded at 70% confluence on 35 mm glass coverslip bottom dishes (MatTek Corporation, Ashland, MA) and allowed to adhere overnight. The medium was then removed and replaced with fresh KGM. At confluence, cells were pre-treated for 1 hour with or without 1 µM of the ULK1/2 inhibitor, MRT68921. The inhibitor was removed, and cells were inoculated with 10^6^ CFU/mL for 2 hours. Ten minutes prior to the conclusion of the 2-hour treatment, cells were treated with 10 µg/mL of tetraethylbenzimidazolylcarbocyanine iodide (JC-1) dye (Invitrogen, Carlsbad, CA). After 10 minutes, cells were washed three times with PBS. Cells were imaged using a Leica SP8 laser scanning confocal microscope with a 63× oil objective. To maintain the cell culture environment during imaging, an environmental chamber was used (Life Imaging Services, Basel, Switzerland). The chamber was kept at 5% CO_2_, 37°C. JC-1-stained monomers were scanned using a 488 nm excitation laser, and JC-1 aggregates (multimers) were scanned using a 561 nm excitation laser. Sequential scanning was performed to prevent spectral crosstalk. Red and green fluorescence were quantified using ImageJ (48).

### Plasmid transfection

For mCherry-GFP-LC3 experiments, hTCEpi cells were seeded at 50%–60% confluence onto 35 mm glass bottom dishes (MatTek Corporation, Ashland, MA) and allowed to adhere overnight. Cells were transfected with a plasmid encoding mCherry-GFP-LC3 (generous gift from Dr. Ciro Isidoro, UNIPO, Novara, Italy) (49) using Lipofectamine 3000 (Invitrogen, Carlsbad, CA) in antibiotic-free basal media. Five micrograms of mCherry-GFP-LC3 plasmid and 10 µL of P3000 reagent were added to 125 µL of Opti-MEM medium (Invitrogen, Carlsbad, CA). Five microliters of Lipofectamine 3000 (Invitrogen, Carlsbad, CA) was then diluted in 125 µL of Opti-MEM medium and added to the DNA mix. The resultant mixture was allowed to incubate for 15 minutes, after which the transfection mix was added directly to hTCEpi cells containing 1 mL of basal media and incubated for 24 hours. The medium was then removed, and cells were cultured in KGM for another 24 hours as indicated. At that time, cells were inoculated with 10^6^ CFU/mL PAO1 for 2 hours. Cells were imaged on a Leica SP8 laser scanning confocal microscope equipped with an environmental chamber as described for JC-1 staining above. A 63× oil objective was used. Fluorophores were scanned using a 488 nm excitation laser (GFP) and a 561 nm excitation laser (mCherry). Image settings were kept constant for infected and non-infected cells. Sequential scanning was performed to prevent spectral crosstalk.

### Gentamicin protection assay

A gentamicin protection assay was used to measure PAO1 internalization in hTCEpi cells and HBECs. Cells were seeded at 50%–60% confluence and allowed to adhere overnight. Cells were then pre-treated for 3 hours with the autophagy flux inhibitor bafilomycin A1 at the concentration of 10 nM or the autophagy stimulator rapamycin at the concentration of 1 µM or for 1 hour with the ULK1/2 inhibitor MRT68921 at the concentration of 1 µM in culture media. Cells were then infected with PAO1 at a cell:bacteria ratio of 1:500. After 2 hours, cells were washed three times in PBS and treated with 200 µg/mL gentamicin in culture media at 37°C for 30 minutes to kill all extracellular bacteria. Cells were then washed three times in PBS and lysed with 0.25% TritonX-100 in PBS for 15 minutes. All samples were serially diluted and plated in triplicate on tryptic soy agar for colony count determination.

### Transmission electron microscopy

hTCEpi cells and HBECs were seeded onto 35 mm glass bottom dishes (MatTek Corporation, Ashland, MA) and allowed to adhere overnight. Cells were cultured in KGM overnight with or without PAO1 for 2 hours. Cells were then fixed with 2.5% glutaraldehyde/0.1 M cacodylate buffer (pH 7.4) for 15 minutes at room temperature. After three washes in 0.1 M sodium cacodylate buffer, cells were post-fixed in 1% osmium tetroxide and 0.8% K3[Fe(CN6)] in 0.1 M sodium cacodylate buffer for 1 hour at room temperature. Cells were rinsed with water and stained with 2% aqueous uranyl acetate overnight. After staining, samples were again rinsed three times with water, dehydrated with increasing concentration of ethanol, infiltrated with Embed-812 resin, and polymerized in a 60°C oven overnight. Blocks were sectioned using a diamond knife (Diatome, Quakertown, PA) on a Leica Ultracut UCT (7) ultramicrotome (Leica Microsystems, Wetzlar, Germany) and collected onto copper grids, which were post stained with 2% uranyl acetate in water and lead citrate. Images were acquired on a JEOL 1400 Plus (JEOL, Peabody, MA) equipped with a LaB6 source using a voltage of 120 kV.

### Enzyme-linked immunoassay

hTCEpi cells were inoculated with 10^6^ CFU/mL of PAO1 for 6 hours with or without the ULK1/2 inhibitor, MRT68921. At the 6-hour time point, medium was then collected and stored at −80^°^C until further analysis. IL-6 and IL-8 cytokine levels were quantified using Quantikine enzyme-linked immunoassays (ELISAs; R & D system, Minneapolis, MN) according to the manufacturer’s instruction. For analysis, samples were first thawed on ice, and appropriate dilutions were determined. Briefly, standards and samples were directly added to wells pre-coated with antibody. After a 2-hour incubation period, wells were washed to remove any unbound protein, and an enzyme-linked polyclonal antibody was added. After a second washing step, samples were incubated with substrate solution. Stop solution was then added, and color development was quantified using a BioTek plate reader (Winooski, VT) at 450 nm. Cytokine concentrations were calculated using a standard curve. All data are representative of three independent experiments. Technical duplicates or triplicates were included for each group.

### Untargeted metabolomics

hTCEpi cells were infected with an overnight culture of PAO1, and metabolites were extracted in 80% methanol with 5 µM heavy internal standard (50). At the end of the treatment period, cell culture supernatants were removed, and ice cold 80% methanol with 5 µM heavy internal standard was added to the cells. After a 5-minute incubation on ice, cells were collected along with 80% methanol. The samples were then subject to 1-minute freeze-thaw cycles oscillating between dry ice and 4°C for a total of 5 times to collect metabolites, followed by centrifugation. The supernatant was then removed and filtered using 0.2 µM centrifugal filters, and liquid chromatography-mass spectrometry (LC-MS) was performed in Sciex QTRAP 6500+ mass spectrometer with EI ion spray in both positive and negative mode. Shimadzu HPLC (Nexera X2 LC-30AD) with analyst 1.7.2 software was coupled to the mass spectrometer. SeQuant ZIC-pHILIC 5 µm polymeric 150 × 2.1 mm polyetheretherketone (PEEK)-coated HPLC column with a temperature of 45°C, injection volume of 5 µL, and flow rate of 0.15 mL/min was used. The mobile phase was solvent A: acetonitrile and solvent B: 20 mM ammonium carbonate with 0.1% ammonium hydroxide and 5 µM of medronic acid. The gradient elution procedure was as follows: 0 minute: 80% B, 20 minutes: 20% B, 20.5 minutes: 80% B, and 34 minutes: 80% for a total of 34 minutes. SCIEX MultiQuant 3.0.3 software was used for the detection and relative quantification of metabolites based on peak area. The peak area of each feature was normalized to the internal standard. MetaboAnalyst 5.0 (McGill University, Montreal, Canada) was used for statistical analysis and data representation. An unpaired ANOVA was used to perform univariate statistical analysis, and Fisher’s least significant difference method correction was applied. An adjusted *P* value/false discovery rate (FDR) <0.05 was considered significant.

### Neutrophil chemotaxis

DMSO-differentiated HL-60 cells were used for this assay. Neutrophil migration was assessed using a commercially available Boyden chamber fluorometric assay (Cell Biolabs, San Diego, CA). In brief, the DMSO-differentiated HL-60 cells were added to the top chamber. In the bottom chamber, supernatants collected from hTCEpi cells 6 hours post infection were added. Cell culture conditions were identical to those used for the ELISA: media-only control, PAO1-infected cells, inhibitor-only control, and PAO1-infected cells treated with the inhibitor. After a 2-hour incubation, the migrated cells in the bottom chamber were lysed, and fluorescence was measured using a BioTek plate reader (Winooski, VT). Data are presented from three independent biological samples per group.

### Statistics

All data are presented as mean ± SD. A two-tailed *t*-test was used to test for differences between two groups. For multiple groups, a one-way ANOVA with an appropriate post hoc multiple comparison test was used. A *P* value < 0.05 is considered statistically significant.

## Data Availability

The metabolomic data set is publicly available at https://utsw.app.box.com/s/ad5qttuldvmdfnapfu5btddnh4upd3aq.

## SUPPLEMENTAL MATERIAL

Figure S1 msphere.00537-24-s0001.tif Role of the ULK1/2 complex in autophagy.

Figure S2 msphere.00537-24-s0002.tif PA induces autophagy in primary cultured HCECs through the attenuation of mTOR signaling.

Figure S3 msphere.00537-24-s0003.tif The inhibition of mTOR requires viable PA.

Legends msphere.00537-24-s0004.docx Supplemental figure legends.

ASM does not own the copyrights to Supplemental Material that may be linked to, or accessed through, an article. The authors have granted ASM a non-exclusive, world-wide license to publish the Supplemental Material files. Please contact the corresponding author directly for reuse.
