# Supplementary figures and images for: Inhibition of Unc-51-like-kinase is mitoprotective during Pseudomonas aeruginosa infection in corneal epithelial cells
Source: mSphere. 2025 Jan 10;10(2):e00537-24. doi: 10.1128/msphere.00537-24 (PMC11852725; doi:10.1128/msphere.00537-24)

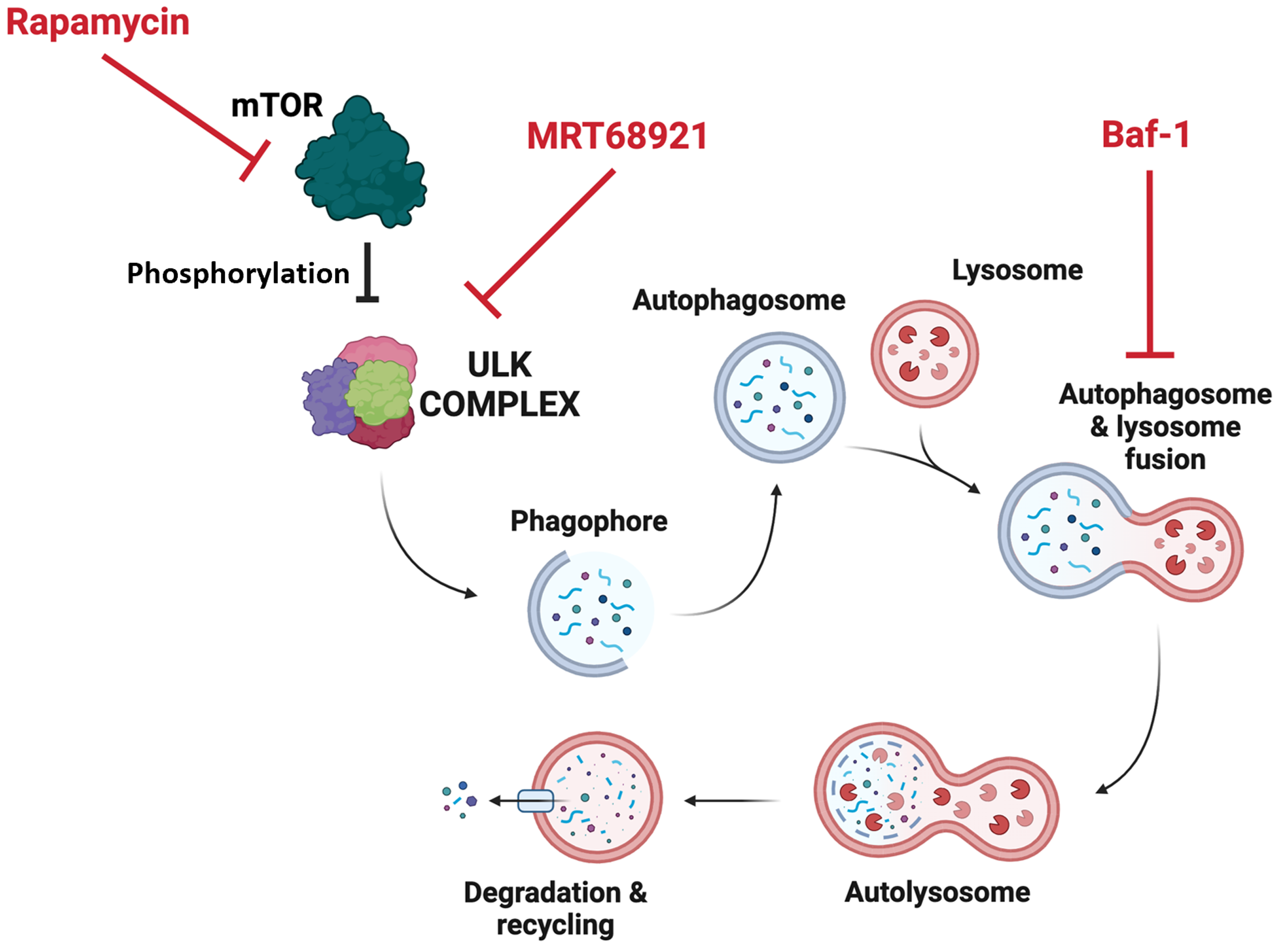

Supplement: Figure S1 — Role of the ULK1/2 complex in autophagy. [file msphere.00537-24-s0001.tif]

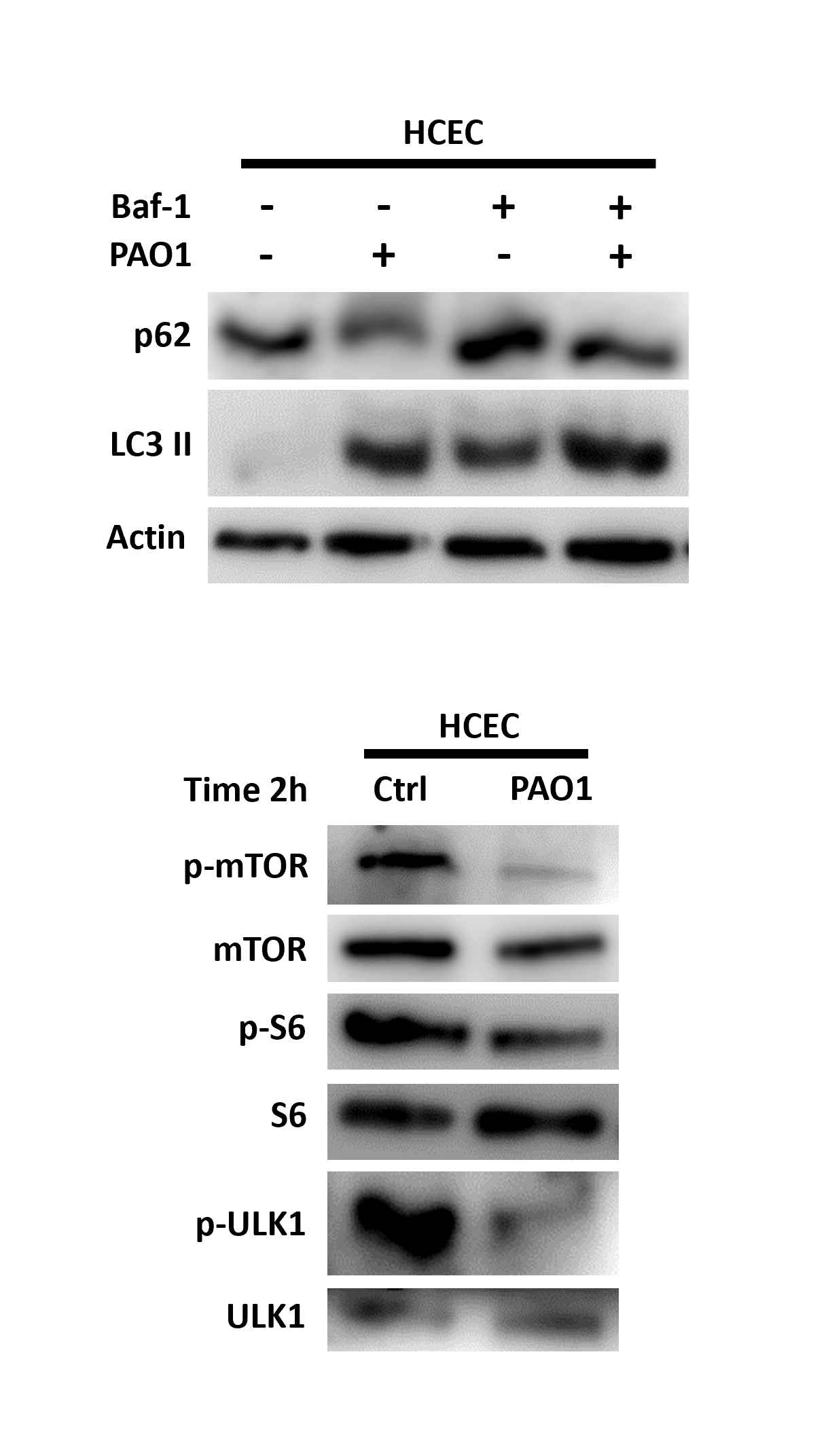

Supplement: Figure S2 — PA induces autophagy in primary cultured HCECs through the attenuation of mTOR signaling. [file msphere.00537-24-s0002.tif]

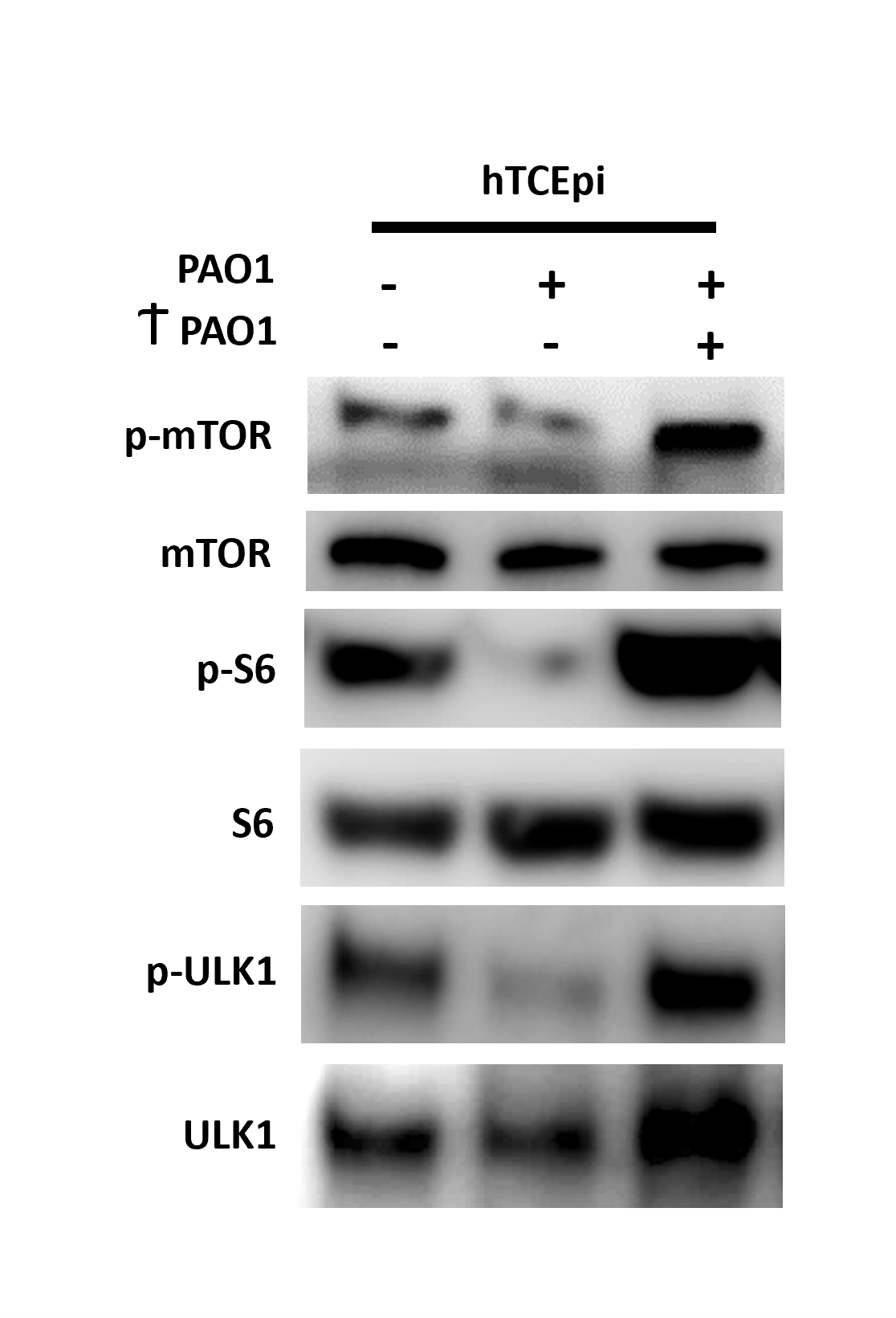

Supplement: Figure S3 — The inhibition of mTOR requires viable PA. [file msphere.00537-24-s0003.tif]
