# Supplementary material for: Inhibition of Unc-51-like-kinase is mitoprotective during Pseudomonas aeruginosa infection in corneal epithelial cells
Source: mSphere. 2025 Jan 10;10(2):e00537-24. doi: 10.1128/msphere.00537-24 (PMC11852725; doi:10.1128/msphere.00537-24)
Supplement: Legends — Supplemental figure legends. [file msphere.00537-24-s0004.docx]

**Supplemental Material**

**Suppl Fig. 1: A schematic illustrating the role of the ULK1/2 complex in autophagy.** Rapamycin blocks mTOR thereby dephosphorylating ULK1/2. This results in the induction of autophagy. In contrast to this, MRT68921 functions to directly inhibit ULK1/2 leading to a block in autophagy. Bafilomycin blocks fusion of the autophagosome and lysosome, thereby preventing degradation of autophagosomal contents. Figure made with BioRender (Toronto, Canada).

**Suppl Fig. 2: PA induces autophagy in primary cultured human corneal epithelial cells (HCECs) through the attenuation of mTOR signaling.** hTCEpi cells were inoculated with bafilomycin-1 (Baf-1) for one hour. Cells were the infected with 10^6^ CFU/ml of PA01 for 2 hours with or without Baf-1. (A) HCEC whole cell lysates were immunoblotted for p62 and LC3-II. Similar to cell line findings, in PA infected cells, there was an increase in LC3 II. In cells treated with Baf-1, accumulation of both p62 and LC3-II was observed. Actin was used as a loading control. (B) Immunoblotting for phosphorylated mTOR, S6, and ULK1. Consistent with hTCEpi cells, mTOR signaling was attenuated in HCECs infected by PA. Total protein levels of mTOR, S6 and ULK1 were used as loading controls. Blots representative of three repeated experiments.

**Suppl Fig. 3: The inhibition of mTOR requires viable PA.** hTCEpi cells were inoculated with 10^6^ CFU/ml of viable or heat-killed PA01 for 2 hours. Immunoblotting was used to measure total and phosphorylated levels of mTOR, S6, and ULK1. Unlike viable bacteria, heat-killed PA did not attenuate phosphorylation of mTOR, S6, or ULK1. Blots representative of three repeated experiments.
